# Validation of Gazelle Microchip Electrophoresis for Premarital Hemoglobinopathy Screening in Türkiye

**DOI:** 10.1002/jha2.70310

**Published:** 2026-06-06

**Authors:** Duran Canatan, Serpil Delibaş, Emel Altunsoy, Elif Gözde Gökkaya, Sultan Aydın, Defne Ay Tuncel, Rucha Natu, Priyaleela Thota, Umut A. Gurkan

**Affiliations:** ^1^ Antalya Bilim University Antalya Türkiye; ^2^ Hemoglobinopathy Diagnosis Thalassemia Center of Mediterranean Blood Diseases Foundation Antalya Türkiye; ^3^ Antalya Genetic Diseases Assessment Center Antalya Türkiye; ^4^ Thalassemia Center, Antalya Education and Research Hospital Health Sciences University Antalya Türkiye; ^5^ Pediatric Hematology Oncology Clinic, Adana City Training and Research Hospital Health Sciences University Adana Türkiye; ^6^ Department of Mechanical and Aerospace Engineering Case Western Reserve University Cleveland Ohio USA; ^7^ Hemex Health Portland Oregon USA

**Keywords:** beta‐thalassemia, hemoglobin variants, hemoglobinopathies, anemia, point‐of‐care

## Abstract

**Introduction:**

Hemoglobinopathies, the most prevalent recessive monogenic disorders globally, encompass thalassemia syndromes and structural hemoglobin variants, affecting approximately 5% of the world's population as carriers, with around 315,000 affected births annually. These conditions are especially common in regions including the Mediterranean Basin, parts of Africa, the Middle East, India, and Southeast Asia. Traditional screeningcomplete blood count (CBC), high‐performance liquid chromatography (HPLC), and capillary electrophoresis (CE)followed by molecular genetic confirmation, requires skilled personnel and advanced facilities, which are often scarce in high‐prevalence, low‐resource areas, rendering them inaccessible to many. Gazelle, a novel, point‐of‐care (POC) microchip‐based cellulose acetate electrophoresis platform, promises an affordable and accessible alternative for detecting common hemoglobin variants.

**Methods:**

This study evaluates Gazelle's performance against HPLC for premarital screening in Antalya, Türkiye, enrolling 616 participants: 516 for screening and 100 known carriers (81 beta‐thalassemia, 19 sickle cell) as controls. Participants underwent CBC, HPLC, and Gazelle testing, with positive results confirmed by beta‐gene sequencing.

**Results:**

Among the 516 screened (55.1% women, 44.9% men, average age 32.75 ± 11.25 years), 16 traits (3.2%) were identified, including 14 beta‐thalassemia, one HbS, and one HbD. Statistical analysis confirmed 100% diagnostic agreement between Gazelle and HPLC.

**Conclusion:**

Gazelle's advantages include rapid results, digital storage, Wi‐Fi connectivity, portability, and printed reports, which make it a transformative tool for identifying hemoglobin variants like HbA, HbS, HbF, and HbA2, and accurately detecting β‐thalassemia carriers in resource‐limited settings. Future efforts will focus on analytically validating Gazelle through evaluations of sample stability, operator variability, and repeatability/reproducibility in clinical settings to strengthen its positioning as a screening platform.

Trial Registration: The authors have confirmed clinical trial registration is not needed for this submission

## 1 Introduction

Hemoglobinopathies represent the most prevalent recessive monogenic disorders globally, encompassing two primary categories: (1) thalassemia syndromes consisting of Alpha (α) and beta (β)‐thalassemia and (2) structural variants of Hb, which include sickle cell disease (HbS), hemoglobin E disease (HbE), hemoglobin C disease (HbC), and Hemoglobin Constant Spring disease (HbCS). With over 5% of the world population carrying hemoglobin variants, globally an estimated 315,000 infants are born with sickle cell disease (SCD) and about 56,000 with clinically significant thalassemia annually [1, 2, 3]. α‐thalassemia results from reduced (α^+^) or absent (α^0^) α‐globin synthesis, whereas β‐thalassemia arises from reduced (β^+^^+^, β^+^), absent (β^0^), or dominant β‐globin production, depending on mutation type. Structural hemoglobin variants occur from amino acid changes in globin chains, altering hemoglobin structure [4, 5]. The disease spectrum ranges from mild non‐transfusion‐dependent thalassemia (NTDT) to severe transfusion‐dependent thalassemia (TDT), depending on mutation combinations.

These disorders exhibit a high prevalence across the Mediterranean Basin, parts of Africa, the Middle East, India, Southeast Asia, Malaysia, and the Pacific Islands [4, 5]. While treatments for thalassemia major remain costly and often inaccessible in developing countries, community‐based prevention—heterozygote detection, education, and prenatal diagnosis—has been effective since the 1970s [6]. Screening for variants in premarital, newborn, or familial testing, and in cases of unexplained anemia or a suggestive family history, is transforming the management of hemoglobinopathies in these regions [7]. For example, in Türkiye, where hemoglobinopathies pose a significant public health challenge [8], the 1993 enactment of the “Fight Against Hereditary Blood Disease” law led to regional thalassemia centers followed by the creation of the Turkish National Hemoglobinopathy Council (TNHC) in 2000, and the launch of Hemoglobinopathy Prevention Program (HPP) in 33 provinces by 2003. By November 2018, this had evolved into the nationwide “Pre‐Marriage Hemoglobinopathy Screening Program,” mandating free premarital testing and offering genetic counseling and IVF support for carrier couples [9, 10]. Screening protocols emphasize rapid, cost‐effective tests followed by molecular confirmation and DNA analysis to ensure diagnostic accuracy [11]. Conventional screening for hemoglobin disorders involves complete blood counts (CBC) and quantitative hemoglobin analysis using high‐performance liquid chromatography (HPLC) or capillary electrophoresis—with molecular genetic testing for confirmation [12]—methods that require specialized laboratories and trained personnel, limiting accessibility in resource‐constrained settings. Recognizing these barriers, the WHO in 2019 designated anemia and SCD as critical diagnostic priorities in low‐ and middle‐income countries, underscoring the need for affordable point‐of‐care (POC) technologies. Current POC options such as HemoCue, AnemoCheck, and lateral flow assays (e.g., Sickle SCAN, HemoTypeSC) address either hemoglobin quantification or variant detection, often requiring multiple platforms for comprehensive evaluation [13, 14, 15]. The Gazelle system, a miniaturized paper‐based microchip electrophoresis platform, overcomes this limitation by enabling rapid, integrated identification and quantification of common hemoglobin variants from a single drop of blood. The Gazelle system integrates detection and quantification of hemoglobin variants from a single blood drop, providing rapid, user‐friendly diagnostics suited for resource‐limited environments [16]. This study aims to evaluate the Gazelle platform for premarital hemoglobinopathy screening in Türkiye, comparing its performance with HPLC and validating results through beta‐gene sequence analysis.

## 2 Materials and Methods

### 2.1 Study Design

This observational validation study, approved by the Medical Faculty of Akdeniz University (Issue: 70904504/334), and the Ministry of Health Pharmaceuticals and Medical Devices Agency (E‐68869993‐511.06.02.99‐1309047), compared the Gazelle microchip electrophoresis to HPLC for premarital hemoglobinopathy screening in Antalya, Türkiye.

### 2.2 Study Population

Couples who applied to the Mediterranean Blood Diseases Foundation (MBDF) Hemoglobinopathy Diagnostic Center for premarital screening were invited to participate in this study. Individuals were excluded if they had any serious or chronic illness requiring urgent medical attention, iron deficiency anemia (serum ferritin < 15 ng/mL), or declined to provide consent. Eligible participants were recruited, screened, and enrolled as per the inclusion and exclusion criteria after providing written or thumb‐impression informed consent, in accordance with standard enrollment procedures. A total of 516 samples were analyzed to assess the performance of Gazelle against HPLC. In addition, 100 control samples from individuals with beta‐thalassemia trait or sickle cell trait were included in the study to validate Gazelle's performance in a population with confirmed hemoglobinopathies.

### 2.3 Study Methods

A 5 mL EDTA venipuncture blood sample was collected from each participant and was utilized for multiple analyses: CBC, HPLC, Gazelle microchip testing, and beta‐gene sequencing for confirmation of positive variants. A sample size of 516 was calculated to detect a 3% carrier prevalence with 95% confidence and 80% power. The CBC (Sysmex XN‐1000) and HPLC (BioRad Variant II) were performed at MBDF. About 20 µL of the sample was used for testing with the Gazelle microchip at Antalya Genetic Diseases Assessment Center (AGTC) following the Gazelle user instructions [15]. Beta‐gene sequencing was conducted for any positive variants to confirm the findings.

### 2.4 Gazelle

A detailed description of design and development of Gazelle was published previously [17]. Briefly, Gazelle is a battery‐/power‐operated miniaturized electrophoresis platform for hemoglobin electrophoresis testing using single‐use disposable cartridge incorporating a cellulose acetate test strip. The different hemoglobin fractions migrate and separate into visible bands on the test strip as HbA (normal), HbS (sickle), HbF (fetal), and Hb (A2/C/E) in about 8 min. The bands are quantified for interpretation and the types, and percentages of hemoglobin are displayed on the screen, with printable results stored in the reader's memory. The reports can be shared through wireless data transfer media using Wi‐Fi or Bluetooth.

### 2.5 Data Analysis

Data analysis was conducted using the software SPSS V29. Sensitivity, defined as the probability that the assay yields a positive result when an Hb variant is present, is calculated as TP / (TP + FN) × 100%, where TP represents true positives, and FN represents false negatives. Specificity, defined as the probability that the assay yields a negative result when an Hb variant is absent, was calculated as TN / (TN + FP) × 100%, where TN represents true negatives and FP represents false positives.

Cohen's Kappa (ĸ) analysis method was used to assess the level of agreement between the Gazelle and the HPLC results. This method was chosen because it evaluates the agreement by adjusting for agreement by chance. Pearson's correlation coefficient (*r*) was calculated for quantitative comparison of the HbA2 results between the Gazelle and HPLC. This statistical test evaluated the strength and direction of a linear association between two methods. The correlation analysis was performed by plotting HbA2 values measured by Gazelle against those measured by HPLC, and the correlation coefficient, along with the corresponding *p*‐value was calculated. In addition, the coefficient of determination (*R* ^2^) was derived to quantify the proportion of variance in HPLC HbA2 values that could be explained by Gazelle measurements. This approach provided an objective assessment of the comparability of HbA2 quantification between the two methods. Further, receiver operating characteristic (ROC) analysis was conducted to analyze the diagnostic performance of Gazelle in comparison with the HPLC performance for HbA2 analysis. The area under the ROC, area under curve (AUC) was calculated as a summary measure of overall diagnostic accuracy, where 0.5 implies no discriminative ability and 1.0 implies perfect discrimination. Logistic regression was used to model the probability of a binary dependent variable (e.g., presence vs. absence of a hemoglobin variant) as a function of the test method—Gazelle or beta‐gene sequencing.

## 3 Results

### 3.1 Participant Characteristics

A total of 616 individuals participated in this study, comprising two distinct groups: a screened group of participants and a control group of known carriers. The screened participants included 516 individuals—284 women (55.1%) and 232 men (44.9%), with an average age of 32.75 ± 11.25 years. Gazelle and HPLC screening identified 16 carriers (3.2%), including 14 individuals with beta‐thalassemia trait, one with HbS (sickle cell trait), and one with HbD. The control group consisted of 100 carriers—81 beta‐thalassemia traits and 19 sickle cell traits. This group included 58 women (58%) and 42 men (42%), with an average age of 26.8 ± 18.6 years. Gazelle was not validated for alpha‐thalassemia or other variants (HbC and HbE) due to low prevalence in the study population. HbD was detected as an abnormal HbS variant since HbD and HbS co‐migrate on Gazelle.

### 3.2 Carrier Detection

**FIGURE 1:**
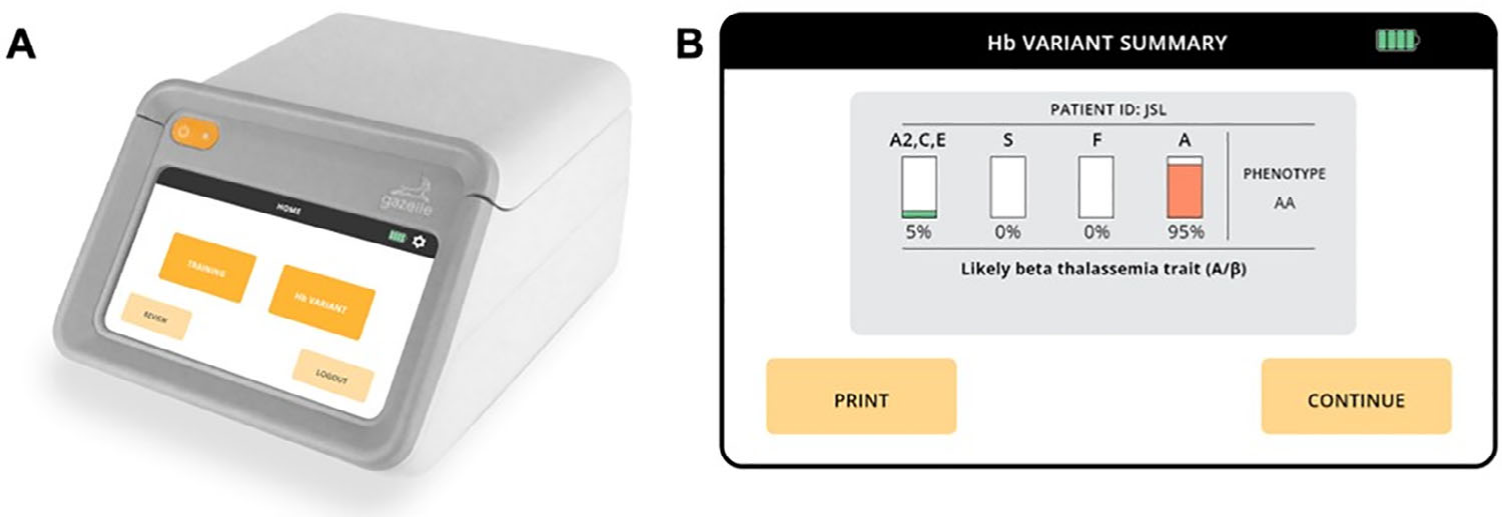
Gazelle, a novel, point‐of‐care (POC) microchip‐based cellulose acetate electrophoresis platform, offers an affordable and accessible alternative for detecting common hemoglobin variants using low volume (∼20 µL) venipuncture blood samples. (A) Device overview. (B) Representative result screen for a beta‐thalassemia trait sample.

The average values for CBC parameters and hemoglobin fractions (HbA1, HbA2, and HbS) measured by both HPLC and microchip electrophoresis (Gazelle, Figure 1) were calculated for 500 healthy participants and 16 identified carriers. The 100 controls were split into beta‐thalassemia (*n* = 81) and HbS (*n* = 19) subgroups. These results are presented in Table 1.

**TABLE 1:** Average values of complete blood count, HPLC, and Gazelle HbA1, HbA2, and HbS results of individuals participating in the study.^a^

Test parameters	Normal group (*n*:500) mean ± SD	Carriers (*n*:16) mean ± SD	Control group thalassemia C. (*n*:81) mean ± SD	Control group HbS carrier (*n*:19) mean ± SD
RBC: 10^6^/µL	4.9 ± 0.6	5.82 ± 0.66	6.3 ± 5.34	5.09 ± 0.85
Hb: gr/dL	14 ± 1.7	12.7 ± 1.79	11.4 ± 1.39	13.4 ± 1.63
Hkt: %	40.7 ± 5.1	37.6 ± 5.15	34.4 ± 4.82	39 ± 4.91
MCV: fL	82.7 ± 9.05	64.5 ± 5.69	61.3 ± 6.21	77.4 ± 7.34
MCH: pg	28.6 ± 2.28	21.8 ± 2.14	20.4 ± 2.17	26.8 ± 2.69
RDW: %	14.6 ± 1.9	15.4 ± 1.07	16.3 ± 2.18	14.4 ± 3.12
WBC: 10^3^/µL	7.58 ± 2.02	8.08 ± 1.84	7.86 ± 2.02	8.54 ± 2.17
PLT: 10^3^/µL	269.2 ± 76.4	295.7 ± 86.08	315.3 ± 115.2	265.5 ± 80.5
HPLC %HbA1	86.2 ± 0.98	79.9 ± 10.2	82.8 ± 2.84	51.4 ± 12.2
HPLC %HbA2	2.8 ± 0.16	4.11 ± 0.8	4.46 ± 0.6	2.3 ± 0.67
HPLC HbS	0	2.3 ± 8.73	0	38.5 ± 13.2
Microchip %HbA1	97.18 ± 6.17	91.1 ± 12.09	95.7 ± 0.75	59 ± 16
Microchip %HbA2	2.4 ± 0.5	3.4 ± 1.65	4.24 ± 0.72	0.15 ± 0.66
Microchip %HbS	0	5.06 ± 13.4	0	39.4 ± 13.6

^a^ Hemoglobin fractions (HbA1, HbA2, and HbS) do not sum to 100% as minor fractions are excluded.

Negative results (i.e., participants with no hemoglobinopathies) were evaluated by verifying the Gazelle and HPLC results against normal CBC findings and the absence of clinical suspicion for hemoglobinopathy. Sequencing was performed only for positive cases due to cost constraints. In the healthy group (*n* = 500), the mean hemoglobin (Hb) level was 14 ± 1.7 g/dL, with an MCV of 82.7 ± 9.05 fL, while the beta‐thalassemia carriers in the control group (*n* = 81) exhibited a lower Hb of 11.4 ± 1.39 g/dL and an MCV of 61.3 ± 6.21 fL, consistent with microcytic anemia.

### 3.3 HbA2 Measurement With Gazelle and HPLC

The Gazelle microchip and HPLC yielded comparable HbA2 values, such as 2.4% ± 0.5% and 2.8% ± 0.16% in the normal group, respectively, and 4.24% ± 0.72% and 4.46% ± 0.6% in the beta‐thalassemia control subgroup. HbA2 cutoffs (3.5% for Gazelle, 3.65% for HPLC) were based on manufacturer guidelines and literature [18, 19].

The Kappa analysis method assessed the agreement between the two devices, revealing 100% consistency in diagnosing hemoglobin variants (*p* < 0.005). The Kappa value of 1.000 indicates perfect compatibility between Gazelle and HPLC across 600 valid cases, underscoring their equivalent diagnostic reliability. The compatibility of HbA2 measurements between Gazelle and HPLC was examined using Receiver Operating Characteristic (ROC) analysis. The AUC for Gazelle HbA2 was 0.796 (cutoff value: 3.5%, sensitivity: 75%, specificity: 100%, 95% CI: 0.75–0.84), while HPLC HbA2 yielded an AUC of 0.942 (cutoff value: 3.65%, sensitivity: 76%, specificity: 100%, 95% CI: 0.91–0.97). These AUC values suggest that both devices perform well in classifying elevated HbA_2_ levels indicative of beta‐thalassemia trait.

### 3.4 Comparison of Diagnostic Performance of Gazelle With Other Methods

Evaluation of the four diagnostic methods—microchip electrophoresis (Gazelle), HPLC, beta‐gene sequencing, and CBC—was performed using Pearson correlation analysis, with results detailed in Table S1. The Spearman rho value of 0.557 between Gazelle and beta‐gene sequencing reflects a moderate positive correlation, indicating that Gazelle measurements align directionally with genetic test outcomes. This correlation was statistically significant (*p* < 0.001), confirming that the compatibility between Gazelle and reference methods is not due to chance. Identical rho values (0.557, *p* < 0.001) were observed for HPLC and CBC relative to beta‐gene sequencing, suggesting that all three methods exhibit a consistent level of agreement with the gold‐standard genetic analysis.

Finally, a direct comparison of diagnostic outcomes across Gazelle, HPLC, and beta‐gene sequencing is presented in Table 2. For distinguishing trait carriers from normal individuals, both Gazelle and HPLC achieved 116 true positives, 500 true negatives, and zero false positives or negatives, resulting in 100% sensitivity, specificity, and accuracy. When compared to genetic analysis, the results remained identical, reinforcing the perfect concordance between the two devices and the reference standard.

**TABLE 2:** The results of microchip electrophoresis (Gazelle) versus HPLC and genetic analysis.

	Trait vs. normal (Gazelle vs. HPLC)	Trait vs. normal (Gazelle vs. genetic analysis)	Trait vs. normal (HPLC vs. genetic analysis)
True positive	116	116	116
False positive	0	0	0
True negative	500	500	500
False negative	0	0	0
Sensitivity	100.00	100.00	100.00
Specificity	100.00	100.00	100.00
Accuracy	100.00	100.00	100.00

Collectively, these statistical findings demonstrate a significant positive relationship between the Gazelle microchip electrophoresis device and established methods (HPLC, CBC, and beta‐gene sequencing). The lack of significant differences in diagnostic performance highlights Gazelle's statistical sufficiency as a standalone tool or in conjunction with other methods for hemoglobinopathy screening in this population.

## 4 Discussion

This study assessed the Gazelle microchip electrophoresis platform for premarital hemoglobinopathy screening in Antalya, Türkiye, demonstrating 100% concordance with HPLC across 616 participants, confirmed by beta‐gene sequencing. Gazelle reliably detected HbS, HbD, and beta‐thalassemia traits, aligning with prior research [15, 16] and extending validation to a high‐prevalence, premarital screening context. Hemoglobinopathy prevalence (3.2%) matched national estimates [8], identifying 14 beta‐thalassemia traits, one HbS, and one HbD carrier. Despite limited HbA2 sensitivity (75% vs. 76% for HPLC), no carriers were missed due to integrated CBC screening. The statistical analyses further validated Gazelle's performance, indicating perfect agreement with HPLC, while the Spearman correlation (*ρ* = 0.557, *p* < 0.001) with beta‐gene sequencing suggests a robust directional alignment with molecular confirmation. These metrics position Gazelle as a viable complementary method to existing protocols, potentially reducing the need for immediate genetic testing. Gazelle can be used along with CBC screening and could potentially ease the testing load on genetic testing laboratories. However, the moderate correlation with sequencing highlights that Gazelle, like HPLC, is not a substitute for molecular diagnostics when definitive mutation identification is required, as emphasized by Canatan et al. [11].

With its distinctive advantages, including ease of use, use of low volume of blood, portability, and rapid results, Gazelle addresses key challenges in hemoglobinopathy screening in low‐resource settings [20], aligning with WHO priorities for primary care diagnostics in low‐ and middle‐income countries [6]. This is particularly relevant in Türkiye, where the HPP has expanded nationwide since 2003, yet challenges persist in rural areas with limited access to centralized testing [9].

Despite its strengths, this study has limitations. The analytical validation of Gazelle to study the effect of sample stability, operator ‐to‐operator variability, repeatability, and reproducibility of the platform would strengthen Gazelle's positioning as an effective screening tool. The sample size (616 participants) collected at a single center in Antalya is modest relative to Türkiye's population, limiting generalizability across diverse geographic and ethnic groups within the country. In addition, Antalya has an established thalassemia infrastructure, which may not fully reflect challenges in more remote areas. The exclusion of participants with serious illnesses also restricts insights into Gazelle's performance in complex clinical scenarios, such as coexisting anemia or pregnancy, where hemoglobinopathy detection can be confounded [7]. Future research should focus on multicenter trials with larger, more diverse cohorts to confirm these findings and assess Gazelle's scalability. Comparative studies incorporating cost‐effectiveness data and real‐world implementation outcomes—particularly in rural Turkish provinces—would strengthen the case for its integration into national screening programs. Exploring Gazelle's performance in neonatal or antenatal screening could also broaden its application, given the distinct hematological profiles in these populations [12]. Enhancing Gazelle's sensitivity for HbA2 detection through technical refinements could align its diagnostic precision more closely with HPLC, further solidifying its role as a frontline tool. Further refinement of Gazelle technology could enhance its applicability in population screening by facilitating the detection of variants D, E, and C.

In conclusion, the Gazelle platform offers a rapid, accurate, and accessible solution for premarital hemoglobinopathy screening in the Turkish population, with performance comparable to HPLC and alignment with genetic confirmation. Its practical advantages make it a promising tool for reducing the burden of thalassemia and SCD in high‐prevalence, low‐resource settings, supporting Türkiye's ongoing efforts to control hereditary blood disorders. With further validation and optimization, Gazelle could redefine community‐based hemoglobinopathy screening worldwide.

## Author Contributions

D.C. and U.A.G. conceptualized the study plans. S.D., E.A., Y.B., E.G.G., S.A., P.T., and D.A.T. assisted with the planning and execution of clinical sample acquisition and testing, including human subject research protocol development, subject recruitment, and blood sample collection and testing. D.C., S.D., E.A., Y.B., E.G.G., S.A., and D.A.T. performed the tests, investigation, data analysis, and visualization. D.C. supervised the work. D.C. drafted the original manuscript, and R.N. compiled the manuscript. All authors reviewed and edited the manuscript.

## Conflicts of Interest

Priyaleela Thota is an employee of Hemex Health Inc. Umut A. Gurkan, Rucha Natu, and Case Western Reserve University have financial interests in Hemex Health Inc. Financial interests include licensed intellectual property, stock ownership, research funding, employment, and consulting. Hemex Health Inc. offers point‐of‐care diagnostics for hemoglobin disorders, anemia, and malaria. The conflicts of interest of Case Western Reserve University employees are overseen and managed by the Conflict of Interests Committee according to a Conflict‐of‐Interest Management Plan. The other authors declare no conflicts of interest.

## Supporting information




**Supporting File 1**: jha270310‐sup‐0001‐tableS1.docx

## Data Availability

The corresponding author of this publication will fulfill all reasonable requests for materials and data.
