# Supplementary material for: Validation of Gazelle Microchip Electrophoresis for Premarital Hemoglobinopathy Screening in Türkiye
Source: EJHaem. 2026 Jun 6;7(3):e70310. doi: 10.1002/jha2.70310 (PMC13241692; doi:10.1002/jha2.70310)
Supplement: Supplementary file 1 — Supporting File 1: jha270310‐sup‐0001‐tableS1.docx [file JHA2-7-e70310-s001.docx]

**Supplementary Table 1:**

**Correlation analysis results of the four different methods.**

| **Correlations** | | | | | | |
| --- | --- | --- | --- | --- | --- | --- |
|  | | | GAZELLE | HPLC | BETA GEN | CBC |
| Spearman's rho  (N=105) | (Gazelle ®) | Correlation Coefficient | 1.000 | 1.000^**^ | .557^**^ | 1.000^**^ |
|  |  | Sig. (2-tailed) | . | . | <.001 | . |
|  | HPLC | Correlation Coefficient | 1.000^**^ | 1.000 | .557^**^ | 1.000^**^ |
|  |  | Sig. (2-tailed) | . | . | <.001 | . |
|  | BETA GEN Sequencing | Correlation Coefficient | .557^**^ | .557^**^ | 1.000 | .557^**^ |
|  |  | Sig. (2-tailed) | <.001 | <.001 | . | <.001 |
|  | CBC | Correlation Coefficient | 1.000^**^ | 1.000^**^ | .557^**^ | 1.000 |
|  |  | Sig. (2-tailed) | . | . | <.001 | . |
| **. Correlation is significant at the 0.01 level (2-tailed). | | | | | | |
